# DFT Study into the Influence of Carbon Material on the Hydrophobicity of a Coal Pyrite Surface

**DOI:** 10.3390/molecules24193534

**Published:** 2019-09-30

**Authors:** Peng Xi, Donghui Wang, Wenli Liu, Changsheng Shi

**Affiliations:** 1 Department of Environmental Engineering, North China Institute of Science and Technology, Beijing 101601, China; 2 China Merchants Ecological Environmental Protection Technology CO., LTD., Chongqing 400060, China; wdh_cmhk@163.com; 3 School of Chemical and Environmental engineering, China University of Mining and Technology (Beijing), Beijing 10083, China; liuwenli@163.com

**Keywords:** carbon materials, hydrophobicity, coal pyrite, symbiosis

## Abstract

From the macroscopic point of view, the hydrophilicity of symbiotic carbon pyrite is weakened overall compared to that of pure pyrite. It is very important to explain the impact of elemental carbon accreted on a pyrite surface on the surface’s hydrophobicity from the perspective of quantum chemistry. To study the influence of adsorbed carbon atoms on the hydrophilicity of a coal pyrite surface versus a pyrite surface, the adsorption of a single water molecule at an adjacent Fe site of a one-carbon-atom-covered pyrite surface and a carbon atom monolayer were simulated and calculated with the first-principles method of density functional theory (DFT). The water molecules can be stably adsorbed at the adjacent Fe site of the carbon-atom-covered pyrite surface. The hybridization of the O 2p (H2O) and Fe 3d (pyrite surface) orbitals was the main interaction between the water molecule and the pyrite surface, forming a strong Fe–O covalent bond. The water molecule only slightly adsorbs above a C atom on the carbon-atom-covered pyrite and the carbon atom monolayer surfaces. The valence bond between the water molecule and the pyrite surface changed from an Fe–O bond to an Fe–C–O bond, in which the C–O bond is very weak, resulting in a weaker interaction between water and the surface.

## 1. Introduction

The flotation desulfurization theory of coal slime is based on the difference in hydrophobicity between coal and a pyrite surface. The properties of a coal pyrite surface are similar to those of coal because of defects in the crystal lattice due to carbon impurities or because of the adsorption of large amounts of carbon on the coal pyrite surface. This leads to the strong floatability of coal pyrite in the flotation process [1]. To efficiently remove coal pyrite during the flotation process and during the subsequent flotation desulfurization, it is very important to study the hydrophobicity of the coal pyrite surface and its formation mechanism. Many scholars have studied the existing forms of carbon in coal pyrite, the relationships between them, and the hydrophobicity of coal pyrite. Shao [2] compared the difference of carbon adsorbed on pyrite and coal pyrite. In that work, they proposed that the floatability of coal pyrite was affected by the symbiotic carbon in the coal pyrite or by other forms of carbon, but they did not analyze the mechanism of the influence. Yu [3] proposed that doped carbon existed in the coal pyrite phase and that it strengthened the floatability of coal pyrite. However, the dissociation of coal and pyrite during the actual flotation process is not complete; more carbon atoms are physically adsorbed on the surface of coal pyrite. Therefore, the effect of a carbon atom’s adsorption on the hydrophobicity of coal pyrite should be considered. Another previous study [4] found that doped carbon and adsorbed carbon were present on the surface of coal pyrite and that these phenomena both strengthened its hydrophobicity, which was determined by using the XRD, SEM-EDS, and contact angle meter methods.

Density functional theory (DFT) is a first-principles method that can give deep insights into the adsorption configurations between adsorbates and a surface. Subsequently, it can reveal the adsorption mechanism of the adsorbates on the surface. Chen et al. [5,6,7] and Li et al. [8,9] investigated the adsorption mechanisms of H_2_O, CaOH, and O_2_ on a pyrite surface. The above studies showed that it is feasible to calculate the adsorption of molecules on a pyrite surface based on DFT. Another previous study [10] found that substituted and adsorbed carbon atoms weakened the adsorption strength of H_2_O at the carbon atom-doping and adsorption position. The adsorption process when the water molecule adsorbs at the adjacent Fe site of a one-carbon-atom-covered pyrite surface or carbon atom monolayer surface has not been studied.

Therefore, in this paper, we calculate the interaction between simple water molecules and a pyrite (001) surface by DFT simulations. The present DFT simulation results are helpful in explaining the hydrophobicity difference between pyrite and coal pyrite both in depth and systematically and do so from a microscopic viewpoint by calculating the water adsorption energy, surface charge transfer, and density of states (DOSs).

## 2. Calculation Methods and Model

### 2.1. Calculation Methods

In the process of structural optimizations, the module of CASTEP was used and the exchange–correlation interaction among electrons was described by the generalized-gradient approximation (GGA)-PW91 [11,12]. The interactions between the ionic cores and the valence electrons (Fe 3d^6^4s^2^, S 3s^2^3p^4^, and C 2s^2^2p^2^) were modelled with ultra-soft pseudopotentials (USP) [13]. We used a cutoff of 350 eV for the plane–wave basis expansion [14] and a Monkhorst–Pack [15,16] k-point sampling density with a 4 × 4 × 4 mesh. The spin polarization and the reciprocal space were included in all calculations. Furthermore, we optimized the carbon atoms and water molecules in a 20 × 20 × 20 Å cubic cell with Brillouin zone sampling restricted to the gamma point in the calculation process. The other parameters were consistent with those in Reference [17].

The strengths of the interactions between the adsorbates (water molecules) and the adsorbent (pyrite surface) were expressed by the adsorption energy (E_ads_) in this paper [18,19,20,21].

### 2.2. Surface Model

In this paper, an FeS_2_ (100) 2 × 2 × 1 supercell surface model cut from an optimized pyrite bulk cell was used as the undoped pyrite surface. The surface has 15 atomic layers and 15 Å vacuum layers, and the bottom 9 atomic layers were fixed.

We calculated the adsorption energy to evaluate the interaction between the adsorbates (carbon atoms or water molecules) and the pyrite surface. The more negative the adsorption energy, the stronger the interaction. The adsorption energies of adsorbates on the pyrite surface were calculated via the following equation:

E_ads_ = E_adsorbate/slab_ − E_adsorbate_ − E_slab_ (1)

where E_ads_ is the adsorption energy, E_adsorbate/slab_ is the energy of the pyrite surface with adsorbed atoms or molecules, E_adsorbate_ is the energy of carbon atoms or water molecules in a cubic cell, and E_slab_ is the energy of the pyrite surface.

## 3. Results and Discussion

### 3.1. Surface Model

**Figure 1:**
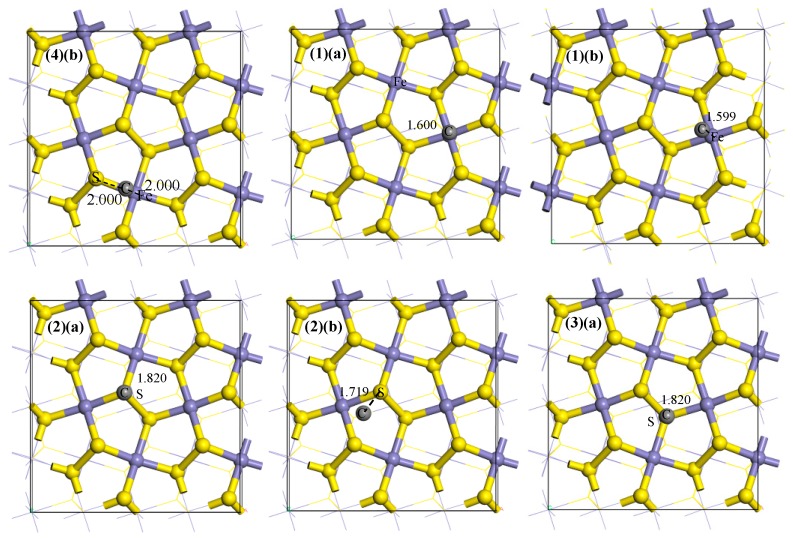

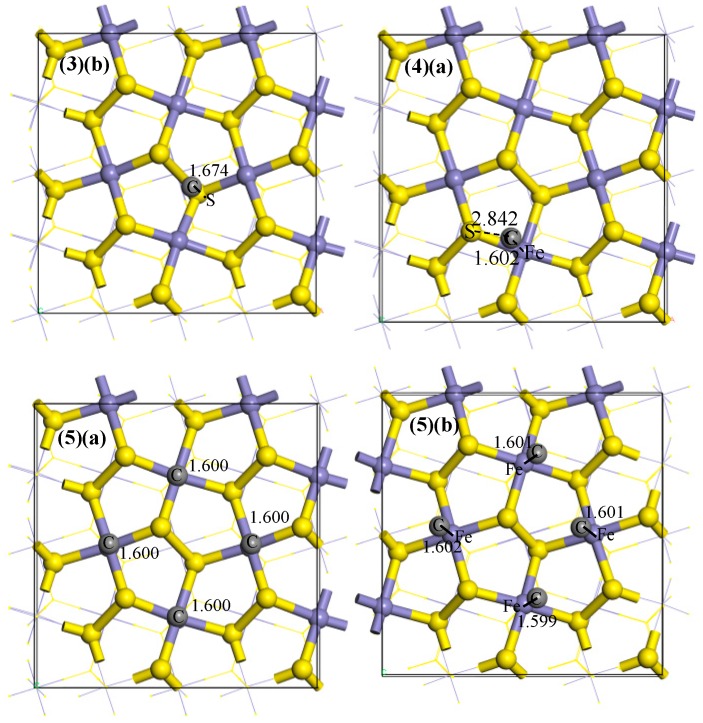
The initial (**a**) and equilibrium (**b**) adsorption models of one carbon atom on the coal pyrite surface for (**1**) F; (**2**) HPS; (**3**) LPS; (**4**) FS; and (**5**) F-4C.

An ideal pyrite surface was cleaved from the optimized unit cell. The coal pyrite surface containing carbon was optimized based on the ideal pyrite surface, in which there were different adsorption sites for the carbon atoms, as shown in Figure 1. Meanwhile, the results of the different adsorption energies are shown in Table 1.

It can be seen from Table 1 that E_ads_ of the four equilibrium configurations are all negative and are roughly −480 kJ/mol for a single carbon atom adsorbed on the pyrite. Additionally, we know that water molecules adsorb most easily above the iron site of the surface, forming two S–H bonds and an Fe–O bond [22]. Therefore, the optimized F model was selected as the subsequent model to study the effect of the adsorbed carbon atom on the hydrophobicity of the pyrite.

### 3.2. Adsorption Energies

**Figure 2:**
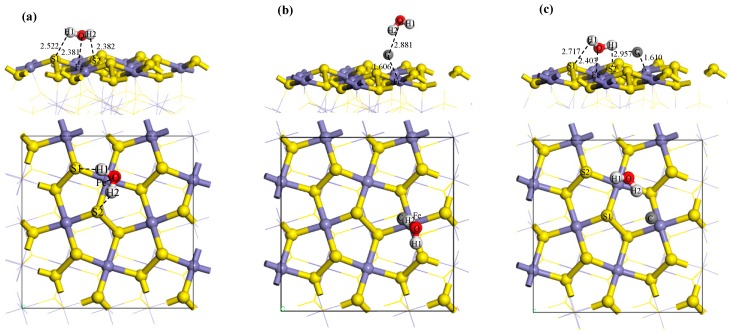

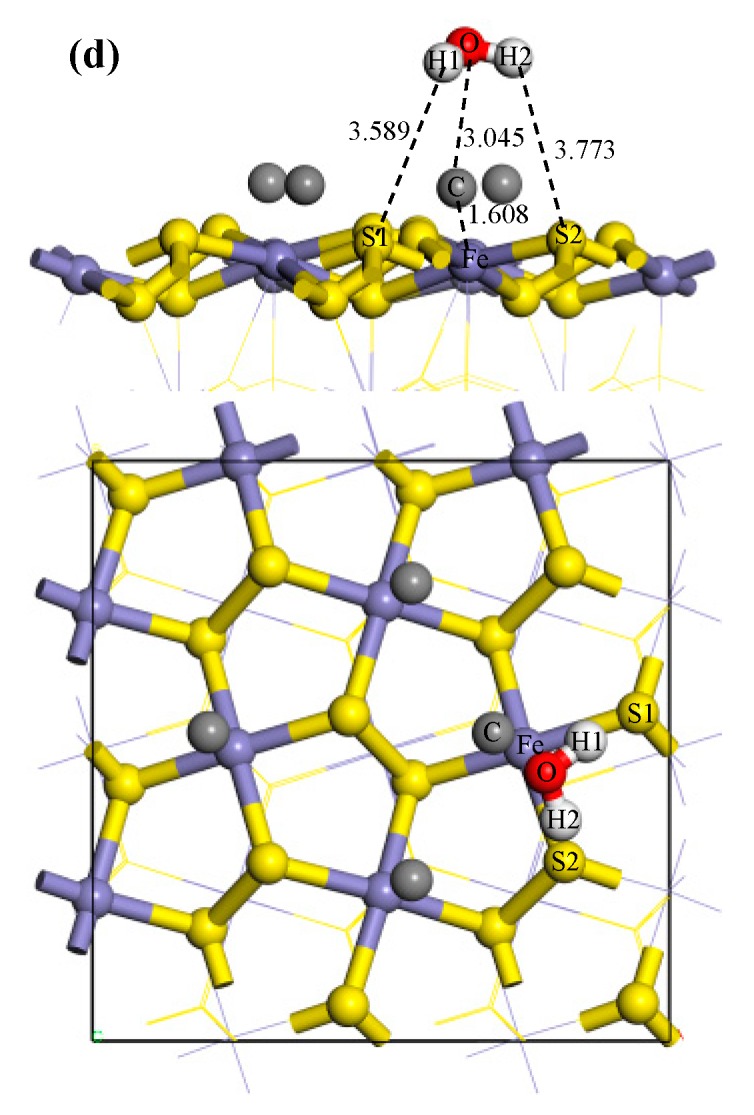
The adsorption configuration of H_2_O on the pyrite surface: (**a**) H_2_O/IS, the adsorption configuration of H_2_O on the ideal pyrite surface; (**b**) H_2_O/AC, the adsorption configuration of H_2_O above the carbon atom of the one-carbon-atom-covered surface; (**c**) H_2_O/AF, the adsorption configuration of H_2_O at the adjacent iron atom on the one-carbon-atom-covered surface; and (**d**) H_2_O/ACM, the adsorption configuration of H_2_O above the carbon atoms of the carbon atom monolayer surface.

For the pure pyrite surface, the equilibrium model predicts that H_2_O adsorbs above the iron atom and forms two high-position S–H bonds. This model is the most stable. To study the effect of carbon atoms on the adsorption of H_2_O on the pyrite surface, the F model from Figure 1 was selected as a typical coal pyrite surface. The water molecule was placed in several locations to study its adsorption. First, the water molecule was placed above the carbon atom (AC), as shown in Figure 2b. To study how the carbon atom affects the adsorption of H_2_O on the adjacent Fe site of the pyrite surface, the water molecule was put at the Fe site adjacent to the carbon atom (AF), as shown in Figure 2c. To study the effect of a carbon atom monolayer on the adsorption of H_2_O on the pyrite surface, the water molecule was placed above the carbon atom monolayer (ACM), as shown in Figure 2d.

Compared with the ideal pyrite surface, when the water molecule adsorbs above the carbon atom, the adsorption energy increases to −20.50 kJ/mol from −51.63 kJ/mol (Table 2). The shows that, after a carbon atom adsorbs on the pyrite surface, the water molecule has more difficulty spontaneously adsorbing above the carbon atom. Thus, the hydrophilicity of the accreted coal pyrite surface is weaker due to the carbon. However, when the water molecule adsorbs on the Fe site adjacent to the carbon atom, the E_ads_ is slightly less negative, which shows that the carbon basically has no effect on the adsorption of the water molecule at the adjacent Fe site. From the macroscopic point of view, the hydrophilicity of coal pyrite locally accreted with coal is weaker than that of the ideal pyrite surface.

For the carbon atom monolayer on the coal pyrite surface, the water molecule adsorption energy E_ads_ is close to 0 kJ/mol, which demonstrates that, after carbon atoms adsorb on the pyrite surface, the water molecule can only slightly adsorb. Thus, a coal pyrite surface accreted by a carbon atom monolayer is nearly hydrophobic.

Based on the above results, we can see that, whether the coal pyrite surface is locally or fully accreted with coal, the hydrophilicity always decreases.

### 3.3. Analysis of Adsorption Configuration and Bonding

**Figure 3:**
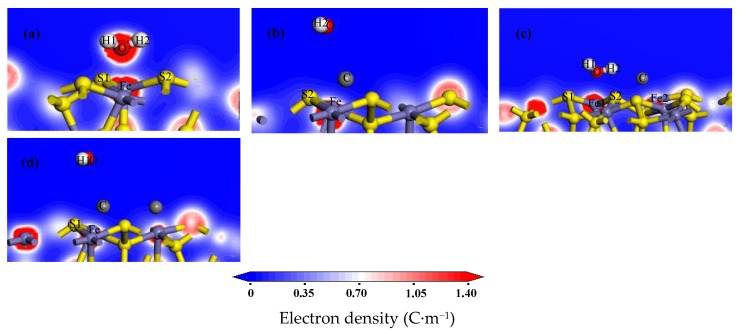
Electron density map after H_2_O adsorption on the pyrite surfaces: (**a**) H_2_O/IS; (**b**) H_2_O/AC; (**c**) H_2_O/AF; and (**d**) H_2_O/ACM.

**Figure 4:**
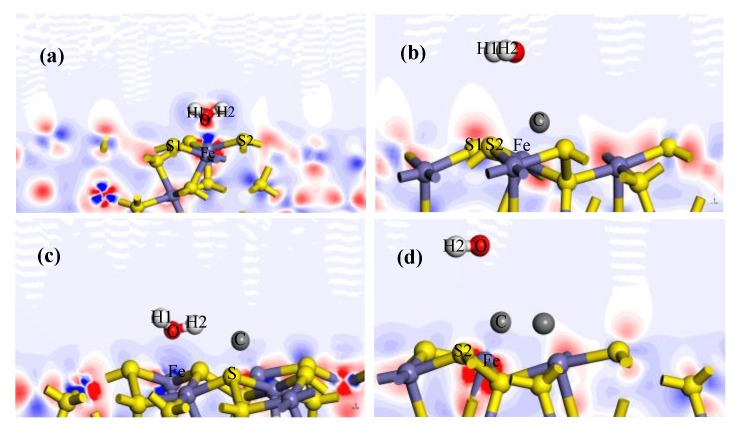
Charge density difference after H_2_O adsorption on the pyrite surfaces: (**a**) H_2_O/IS; (**b**) H_2_O/AC; (**c**) H_2_O/AF; and (**d**) H_2_O/ACM.

The adsorption configurations of water molecules on the pyrite surface are presented in Figure 2a–d. Meanwhile, the electron density and charge density differences of H_2_O on the pyrite surface are shown in Figure 3 and Figure 4. Map slices of electron density and charge density differences are located between water molecules and the pyrite surface. The density of the electron cloud and the ability to gain electrons are also represented by the depth of color in the electron density map. Table 3 shows the bond Mulliken population and length between the water molecules and the pyrite surface. The larger the values of the bond Mulliken population, the stronger the covalent interaction between the water molecule and the pyrite surface [23].

The oxygen atom and hydrogen atoms of the water molecule respectively bond with an iron atom and sulphur atoms of the pyrite surface after H_2_O adsorption, as shown in Figure 2a. From the results of the bond Mulliken population in Table 3, we see that a strong covalent bond (Fe–O) forms between the oxygen atom of the water molecule and the iron of the surface (bond population = 0.10). Meanwhile, the hydrogen atoms of the water molecule bond with the sulphur atoms of the surface to form a weak hydrogen bond, with populations of 0.01 or 0.02. As shown in Figure 3a and Figure 4a, the charge density is higher and there is obvious charge transfer between the oxygen and iron atoms.

Similar to the above results, when the water molecules adsorb at the Fe site adjacent to the carbon atom, the oxygen atom and hydrogen atoms of the water molecule are also bonded with the iron atom and sulphur atoms of the pyrite surface, respectively. The Fe–O bond population is 0.09, and the electron density distribution and charge density difference are shown in Figure 3c and Figure 4c. The same conclusion is made by regarding the adsorption energies, as shown in Table 2: E_ads_(IS) = −51.63 kJ/mol and E_ads_(AF) = −48.09 kJ/mol.

Figure 2b shows the configuration of a water molecule adsorbed to a carbon atom on the pyrite surface. The iron atom of the pyrite surface is bonded with the adsorbed carbon atom above the pyrite surface to form a very strong covalent bond (Fe–C), the population of which is 0.87. Meanwhile, the C–O bond formed between the oxygen atom of the water molecule and the carbon atom is very weak, the population if which is almost zero. It can be clearly seen that there is a higher charge density and an obvious charge transfer between the carbon atom and the iron atom, as shown in Figure 3b and Figure 4b. Similar to these results, when a water molecule adsorbs above a carbon atom monolayer surface, a strong Fe–C covalent bond and a weak C–O bond are formed. The adsorption energies show the same conclusion: E_ads_(AC) = −20.50kJ/mol and E_ads_(ACM) = −6.82kJ/mol.

These results indicate that the adsorption of a water molecule on the pyrite becomes less stable when the pyrite surface is accreted with coal.

### 3.4. Analysis of the Density of States (DOS) and Charge Transfer

**Figure 5:**
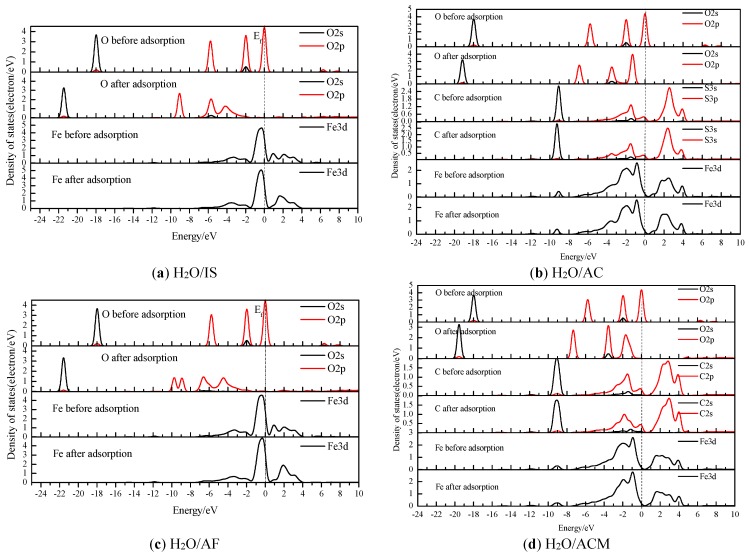
Density of states (DOS) of atoms before and after H_2_O adsorption on different pyrite surfaces.

The interaction between H_2_O and the coal pyrite is mainly through the O atom of the water and the C or Fe atoms of the surface. The Fermi level (Ef) is 0 eV [24]. The DOS results for H_2_O/IS and H_2_O/AF are shown in Figure 5a,b, respectively, and the DOS results for H_2_O/AC and H_2_O/ACM are shown in Figure 5c,d, respectively.

The Mulliken charge populations of the water molecule and surface refer to the loss and transfer of electrons and are shown in Table 4, Table 5, Table 6 and Table 7. BA and AA means before adsorption and after adsorption, respectively.

As in the case of the ideal surface DOS in Figure 5a [17], the one-carbon-atom-covered surface with water adsorbing the adjacent to Fe site, Figure 5b, shows a strong hybridization between the Fe (surface) and O (water molecule) from about −8.2 eV to −1.6 eV. Furthermore, the antibonding state from approximately 0.4 eV to 2.5 eV is also quite weak, as shown in Figure 5c. The O charge increases since the O 2p orbitals lose about 0.20 e, and the Fe charge reduces since it gains some electrons (Table 4 and Table 6). The above results demonstrate that the hybridizations of O 2p and Fe 3d orbitals are the main interaction (H_2_O/IS and H_2_O/AF), which is consistent with the results of the bond population calculation.

For H_2_O/AC and H_2_O/ACM, the bonding and the antibonding states between Fe 3d and C 2p are quite strong. However, the bond C 2p–O 2p is seemingly weaker than the Fe 3d–C 2p interaction. The O charge increases from the O 2p losing approximately 0.06 electrons (Table 5 and Table 7). The C charge that bonds to the O increases, owing to the C 2p losing some electrons. Additionally, the Fe charge reduces due to the addition of electrons. These results agree well with the bond Mulliken population results between the Fe (surface) and C (0.88) and the Mulliken population results between C and O (water molecule) (about −0.01), as shown in Table 3. These results indicate that the covalent overlap between C and O (water molecule) was weak to some extent and, thus, that the symbiotic carbon prevented the adsorption of a water molecule on the surface of pyrite to a certain extent.

## 4. Conclusions

The interaction between a water molecule and coal pyrite, where pyrite was adsorbed by one or a few carbon atoms, was studied using DFT calculations. As with the ideal pyrite surface, a single H_2_O can stably adsorb adjacent to the Fe site on a surface with a single carbon atom. The hybridization of the O 2p (H_2_O) and Fe 3d (pyrite surface) orbitals was the main interaction between the single water molecule and the pyrite surface, forming a strong Fe–O covalent bond. The water molecule could only slightly adsorb on the carbon-atom-covered pyrite surface (above C) and the carbon atom monolayer surface. The valence bond changed from an Fe–O bond to an Fe–C–O bond, in which the C–O bond was very weak.

Furthermore, the hydrophilicity of the coal pyrite surface accreted became weak and almost hydrophobic compared to an ideal pyrite surface. From the macroscopic point of view, the hydrophilicity of symbiotic carbon pyrite was weakened overall. The hydrophilicity difference between the pyrite and the coal pyrite was clarified from the molecular level based on the insight of the symbiotic relationship between coal and pyrite.

## Figures and Tables

**Table 1:** The adsorption energy, E_ads_, for carbon atoms on the pyrite surface.

Adsorption Configuration of Carbon Atom	E_ads_ (kJ/mol)
F	−472.74
HPS	−477.10
LPS	−482.53
FS	−478.97
F-4C	−1862.33

Note: For convenience, the following symbols are used to express the different configurations for the initial adsorption site of the carbon atom. F indicates that the site is above the Fe atom. HPS indicates that the site is above the high-position S atom. LPS indicates that the site is above the low-position S atom. FS indicates that the site is above the Fe–S bond. F-4C indicates that the sites for four carbon atoms are above the Fe atom.

**Table 2:** E_ads_ of H_2_O on different pyrite surfaces.

Adsorption Configuration	E_ads_/(kJ/mol)
H_2_O/IS	−51.63
H_2_O/AC	−20.50
H_2_O/AF	−48.09
H_2_O/ACM	−6.82

**Table 3:** Bond Mulliken population and length after H_2_O adsorption on different pyrite surfaces.

Adsorption Model	Bond	Population	Length
H_2_O/IS	Fe–O	0.10	2.381
	H1–S1	0.01	2.552
	H2–S2	0.02	2.382
H_2_O/AC	Fe–C	0.87	1.606
	C–O	−0.07	2.881
H_2_O/AF	Fe1–O	0.09	2.403
	H1–S1	0.00	2.717
	H2–S2	0.00	2.957
H_2_O/ACM	Fe–C	0.88	1.608
	C–O	−0.01	3.045

**Table 4:** Mulliken charge populations of atoms for H_2_O/IS.

Atomic Label	Adsorption Status	Ion Spin	s	p	d	T	Charge/e
Fe	BA	up	0.17	0.22	3.57	3.97	0.07
		down	0.17	0.22	3.57	3.97	
	AA	up	0.17	0.22	3.56	3.95	0.10
		down	0.17	0.22	3.56	3.95	
O	BA	up	0.95	2.58	0.00	3.53	−1.05
		down	0.95	2.58	0.00	3.53	
	AA	up	0.94	2.49	0.00	3.42	−0.85
		down	0.94	2.49	0.00	3.42	

**Table 5:** Mulliken charge populations of atoms for H_2_O/AC.

Atomic Label	Adsorption Status	Ion Spin	s	p	d	T	Charge/e
Fe	BA	up	0.16	0.27	3.51	3.94	0.12
		down	0.16	0.27	3.51	3.94	
	AA	up	0.16	0.27	3.51	3.93	0.14
		down	0.16	0.27	3.51	3.93	
	BA	up	0.91	1.67	0.00	2.08	−0.15
C		down	0.91	1.67	0.00	2.08	
	AA	up	0.90	1.20	0.00	2.10	−0.21
		down	0.90	1.20	0.00	2.10	
O	BA	up	0.95	2.58	0.00	3.53	−1.05
		down	0.95	2.58	0.00	3.53	
	AA	up	0.93	2.56	0.00	3.49	−0.98
		down	0.93	2.56	0.00	3.49	

**Table 6:** Mulliken charge populations of atoms for H_2_O/AF.

Atomic Label	Adsorption Status	Ion Spin	s	p	d	T	Charge/e
Fe	BA	up	0.17	0.22	3.57	3.96	0.08
		down	0.17	0.22	3.57	3.96	
	AA	up	0.16	0.22	3.55	3.93	0.14
		down	0.16	0.22	3.55	3.93	
O	BA	up	0.95	2.58	0.00	3.53	−1.05
		down	0.95	2.58	0.00	3.53	
	AA	up	0.93	2.48	0.00	3.41	−0.81
		down	0.93	2.48	0.00	3.41	

**Table 7:** Mulliken charge populations of atoms for H_2_O/ACM.

Atomic Label	Adsorption Status	Ion Spin	s	p	d	T	Charge/e
Fe	BA	up	0.15	0.27	3.51	3.93	0.14
		down	0.15	0.27	3.51	3.93	
	AA	up	0.15	0.28	3.52	3.95	0.11
		down	0.15	0.28	3.52	3.95	
	BA	up	0.91	1.14	0.00	2.06	−0.11
C		down	0.91	1.14	0.00	2.06	
	AA	up	0.91	1.14	0.00	2.05	−0.11
		down	0.91	1.14	0.00	2.05	
O	BA	up	0.95	2.58	0.00	3.53	−1.05
		down	0.95	2.58	0.00	3.53	
	AA	up	0.94	2.56	0.00	3.50	−1.00
		down	0.94	2.56	0.00	3.50	
